# Epoxy and Bio-Based Epoxy Carbon Fiber Twill Composites: Comparison of the Quasi-Static Properties

**DOI:** 10.3390/ma16041601

**Published:** 2023-02-14

**Authors:** Carlo Boursier Niutta, Raffaele Ciardiello, Andrea Tridello, Davide S. Paolino

**Affiliations:** Department of Mechanical and Aerospace Engineering, Politecnico di Torino, Corso Duca degli Abruzzi 24, 10129 Turin, Italy

**Keywords:** bio-composites, epoxy composites, mechanical comparison, material modelling

## Abstract

In recent years, interest in sustainability has significantly increased in many industrial sectors. Sustainability can be achieved with both lightweight design and eco-friendly manufacturing processes. For example, concerns on the use of thermoset composite materials, with a lightweight design and a high specific strength, have arisen, since thermoset resins are not fully recyclable and are mainly petrol based. A possible solution to this issue is the replacement of the thermoset matrix with a recyclable or renewable matrix, such as bio-based resin. However, the mechanical properties of composites made with bio-based resin should be carefully experimentally assessed to guarantee a safe design and the structural integrity of the components. In this work, the quasi-static mechanical properties of composite specimens (eight layers of carbon fiber fabric) made with commercially available epoxy and a bio-based epoxy resins (31% bio content) are compared. Tensile tests on the investigated resins and tensile, compression, shear and flexural tests have been carried out on composite laminates manufactured with the two investigated resins. A finite element model has been calibrated in the LS-Dyna environment using the experimentally assessed mechanical properties. The experimental results have proven that the two composites showed similar quasi-static properties, proving that bio-based composite materials can be reliably employed as a substitute for epoxy resins without affecting the structural integrity of the component but lowering their carbon footprint.

## 1. Introduction

In recent years, the interest towards sustainability in industry has significantly grown, due to increasing concerns about pollution and the consequent climate change. Accordingly, in many industrial sectors, such as the automotive and aerospace sectors, the regulations in terms of emissions are becoming ever stricter [1,2], thus boosting the use of lightweight materials and, in general, encouraging lightweight and sustainable design. Composites are the ideal “lightweight materials”, since they are characterized by a high specific strength compared to metallic materials. For this reason, composite materials are gradually replacing metallic materials in many applications, ensuring a significant weight reduction without compromising the structural integrity of the structures.

Among all the composite materials, thermoset composites are the most used for structural applications. They are commonly designed in different configurations (with unidirectional fiber or fabrics) and with different reinforcements, mainly glass fiber or carbon fiber, to achieve the mechanical properties required by the specific structural application. Their use in the automotive sector is rapidly increasing, especially for electric vehicles, and they are widely used in the aerospace sector. Although these materials can reduce weight and emissions, there are many concerns about their full sustainability, because thermoset resins are not fully recyclable and are mainly petrol based. The manufacturing process of thermoset composites is therefore not fully sustainable and eco-friendly. For example, according to [3], epoxy resin synthesis has a significant environmental impact, since it negatively contributes to the total carbon footprint and accordingly to the greenhouse emissions of the automotive industry. Moreover, regulations [4,5] in terms of pollution and environment have been imposed to reduce the use of petrol-based and non-renewable components [6,7]. A possible solution to this issue is the replacement of the thermoset matrix with recyclable or renewable matrices. However, thermoplastic composites cannot be an effective solution since they are characterized by lower mechanical properties, thus not ensuring the required level of strength and safety in most structural applications [8]. Furthermore, the glass transition and melting temperatures of the thermoplastic polymers can be a barrier to some industrial applications. In recent years, polymer industry and research have concentrated their efforts into finding specific formulations able to guarantee good mechanical performance and chemical and thermal stability as composite matrices [9,10]. These two studies concluded that 80% of the polymer materials used to fabricate composites are obtained from non-renewable fossil resources. Thus, the replacement of thermoset matrices with renewable bio-based parts represents an effective solution for contributing to decarbonization goals. Other strategies to lower the environmental impact of materials consist of adding waste to the matrix as fillers [11,12,13].

Different works that have compared the mechanical properties of bio-based resins with those of epoxy resins have been reported in the literature to show whether the mechanical properties are comparable. Derahman et al. [6] compared the mechanical properties of composite materials fabricated with a bio-based resin (obtained with Jatropha seed) produced by the authors and synthetic epoxy resin. The authors studied the mechanical properties of composite laminates made with a completely bio-based matrix and two different concentrations that use epoxy resin with 25% and 50% bio content. The mechanical tests showed that the tensile and flexural properties were not comparable to an epoxy resin and the addition of the bio content was detrimental for the epoxy resin as well. Gour et al. [14] added six different percentages of two bio-based resins (cardanol based) to an epoxy resin both produced by the company Cardolite. The two bio-resins added to the epoxy resin presented different epoxy equivalent weights (EEW), that once mixed with the epoxy resin led to different chemical structures. The authors studied the mechanical and chemical behavior of these compounds (5%, 10%, 15%, 20%, 25% and 30%). Izod impact tests showed that the addition of 30% bio-resin led to an increase in the absorbed energy of 120% and 180% (for the bio-resin with higher EEW) compared to the neat one. On the other hand, the tensile tests showed the highest increase in the ultimate load of 7% for the epoxy modified with 30% bio-based resin with the lower EEW, while the addition of the resin with a higher EEW was detrimental to the tensile properties. Terry and Taylor [7] showed that fully bio-based monomers can be deleterious to the mechanical properties and glass transition temperature (Tg), which decreased with the addition of bio content, as also shown by Derahman et al. [6]. For this reason, the use of partially bio-based epoxies can offer a valid alternative to lowering the carbon footprint for vacuum-infused composites in different industries such as automotive, marine, aerospace and wind energy. Terry and Taylor [7] carried out a comprehensive study on the mechanical properties on the addition of bio-content to commercially available epoxy resins. The study showed that the most promising systems that contained 28%, 36% and 43% bio content had good values of strength, stiffness and toughness, comparable with the corresponding epoxy properties. Nikafshar et al. [15] reported the mechanical properties of two vanillin-based epoxy resins (renewable aromatic compound) by comparing them to a traditional epoxy resin, diglycidyl ether bisphenol A (DGEBA). The motivation of their work was related to the high environmental impact of DGEBA resin, in particular of bisphenol A [16]. They illustrated that a compound prepared with a traditional hardener modified with the same vanillin compound presented enhancements of ~8% in tensile strength and impact Izod strength compared to the traditional DGEBA. However, a decrease in the glass transition temperature of 48 °C was reported, which could be limiting for many industries. Ma et al. [17] used itaconic acid as an alternative feedstock to prepare bio-based epoxy resins. They showed that all the bio-based resins prepared with itaconic acid presented higher mechanical and thermal properties compared to DGEBA. Furthermore, they show that these bio-based resins could reach higher glass transition temperatures of ~135°.

However, although the mechanical properties of these resins seem promising, more research activities are necessary to compare the mechanical properties of composites made with bio-based resins to those of commonly used epoxy resin composites to guarantee a safe design and the structural integrity of the components. Furthermore, the possibility to use these resins for vacuum infusion processes should be also properly investigated, since the preliminary studies [6,7] reported an increase in the viscosity that could limit the use of vacuum infusion. The mechanical characterization of bio-based composite laminates is therefore fundamental and of utmost interest among universities and industry, since it represents the most promising way to lower the carbon footprint for manufacturers by using the same production technology. Few works in the literature aim to compare the mechanical properties between composite laminates made with epoxy and bio-based epoxy resins. Yashas Gowda et al. [18] compared the mechanical properties of a laminate made of hybrid basalt and areca fibers. Tensile tests and flexural tests showed a decrease of ~5% for the tensile and flexural strengths and tensile and flexural moduli for the composites prepared with bio-based epoxy resin.

In the present paper, the quasi-static mechanical properties of composite specimens made with an epoxy resin and a bio-based epoxy resin commercially available are compared. Even if there is an increasing amount of research on this fundamental subject in the literature, a detailed comparison of the quasi-static mechanical properties of epoxy and bio-based commercially available composites is of utmost interest among not only universities but also industry. This paper aims to fill this gap of knowledge, thus focusing on the structural integrity of composite components. Carbon fiber fabric composite laminates (eight layers) with commercially available epoxy and an epoxy resin with 31% bio content were manufactured. Tensile tests on the investigated resins were carried out. Thereafter, tensile, compression, shear and flexural tests were carried out on specimens cut from composite laminates manufactured with the two investigated resins. The experimental mechanical properties were properly selected, since they allow to calibrate a numerical model in the LS-Dyna environment that can be used also for the simulation of complex components or structures. The paper therefore also addresses the simulation of the mechanical response of composite materials, which is a debated subject in the literature but is vital for assessing the structural integrity of the components that are to be designed. The numerical model developed in the paper has been verified using the experimental data of both epoxy and bio-based composites and can be reliably used in the design of composite structures.

## 2. Materials and Methods

This section focuses on the description of the tested resins and composite laminates and of the experimental activity. In Section 2.1, the properties of the epoxy and the bio-based resins are reported, also describing the procedure for manufacturing the composite laminates. In Section 2.2, the configuration of the tensile tests on the two investigated resins and on composite specimens is described. The compression, shear and flexural testing setup are analyzed in Section 2.3, Section 2.4 and Section 2.5, respectively. Finally, in Section 2.6, the numerical models are presented.

### 2.1. Composite Materials

Composite laminates made of carbon fibers were prepared with two different epoxy resins: a traditional commercially available epoxy resin IN2 (EasyComposite Ltd., Rijen, The Netherlands) [19] and an epoxy resin with 31% of bio content IB2 (EasyComposite Ltd., UK) [20]. The bio-content of the bio-based resin is due to the plant-derived glycerol in place of petroleum-based propylene. The epichlorohydrin of the bio-based epoxy resin is manufactured using renewable plant-based glycerol in place of petroleum-based propylene. The technical datasheet does not report the exact plants that the glycerol was extracted from. However, Gerber et al. [21] reports that glycerol is mainly extracted from crop plants. Miyuranga et al. [22] illustrated that glycerol can be extracted from various plant oils. Soybean oil, palm oil and coconut oil can produce glycerol through a process called transesterification. In this process, an alcohol, such as methanol or ethanol, is used to induce a reaction between the plant oil to produce glycerol and fatty acid methyl or ethyl esters (biodiesel).

The two resins present similar mechanical properties that are reported in Table 1.

The fabric (Pyrofil TR30S 3k) is a 210 g 2 × 2 carbon twill (Mitsubishi Corporation, JP) with 3000 filaments per tow (3k). The warp and weft of the fabric are 5.4 and 5.1 per cm, respectively. The datasheet reports also a pre-coating of polyester to allow easy handling of the fabric. The fabric is characterized by a maximum tensile strength of 4410 MPa, a tensile modulus of 235 GPa and a maximum elongation of 1.8%. The woven fabric has a length of yarn of about 8 mm, while the width and the height of the tows are about 1.98 mm and 0.12 mm, respectively, according to the supplier datasheet.

Composite laminates, made of eight layers, were obtained with the vacuum infusion technique. The two resins were degassed in a vacuum chamber before the infusion and cured for 24 h at room temperature. Thereafter, composite laminates prepared with IN2 resin were post-cured in the oven at 100 °C for 3 h. On the other hand, composite laminates prepared with IB2 resin were post-cured in the oven at 80 °C for 8 h. By using these manufacturing parameters, IN2 and IB2 resins presented glass transition temperatures of 95 °C and 85 °C, respectively. The resins are two commercial resins. The provider reports that the bio-epoxy resin can be used in the same environment and for the same applications as epoxy resins. The only limitation is related to a slightly lower, 85°, glass transition temperature that however fits with many industrial sectors, such as aerospace and marine industries. The resulting average thicknesses of the plates were 1.96 mm for the epoxy-based composite and 2.1 mm for the bio-based composite. Therefore, a lower fiber volume fraction was obtained in the bio-based composite, equal to 0.51, while that of the epoxy-based composite was equal to 0.535.

Tensile, compressive, shear and flexural specimens were cut by using a waterjet machine. For each type of test and material, two specimens were tested.

### 2.2. Tensile Tests

In this section, the configuration for tensile tests on the epoxy and the bio-based resin (Section 2.2.1) and on the composite specimens obtained with the investigated resins (Section 2.2.2) are described.

#### 2.2.1. Tensile Tests on Resin

**Figure 1:**
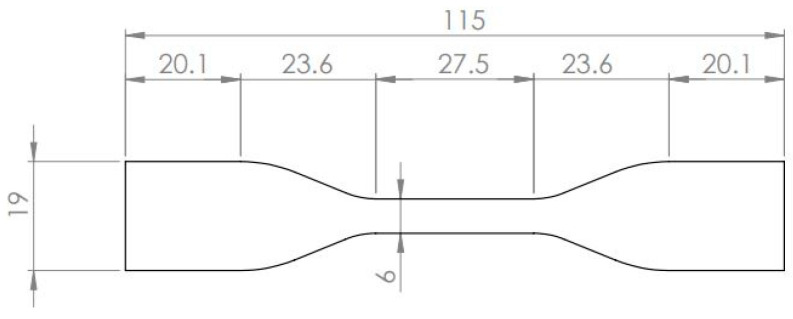
Geometry of the resin specimens.

Firstly, tensile tests on the resins were carried out to verify the mechanical properties provided by the material supplier. The specimen, whose geometry has been defined according to the ASTM D638–14 Standard [23] and is shown in Figure 1, was manufactured by casting the resin in a silicon mold.

Tensile tests were carried out by using a Zwick testing machine equipped with a 5 kN load cell, by imposing a crosshead displacement of 1 mm/min.

#### 2.2.2. Tensile Tests on Composite Specimens

Tensile tests were thereafter carried out on composite specimens manufactured with the two investigated resins. An Instron 8801 testing machine, equipped with a 100 kN load cell, was used for the tests by imposing a crosshead displacement of 1 mm/min. The strain during the tests was measured with the Digital Image Correlation technique (DIC). Random speckle was sprayed on the specimen surface to track surface displacements and calculate the material deformation. The analysis of the results is mainly governed by the subset size parameter and by the step size parameter, which define the size of the virtual mesh of the squared subset and the distance between the subset centers, respectively [24]. In this work, the subset size was equal to 33, while the step size was equal to 8, which is less than 1/3 of the subset size, according to [25].

Rectangular cross-section specimens with a nominal width of 25 mm and a length of 250 mm were tested in accordance with ASTM D3039 [26]. The actual width and thickness of each specimen were measured with a digital caliber (resolution of 0.01 mm) to compute the applied stress more accurately.

**Figure 2:**
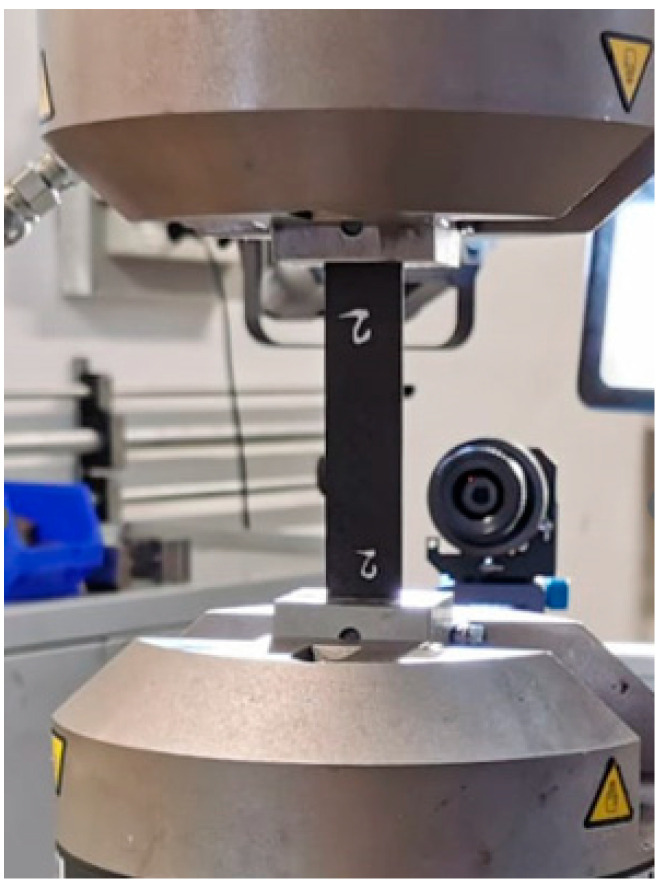
Tensile tests on composite laminates: testing configuration.

Figure 2 shows the tensile test setup. The specimens and the testing machine, together with the DIC system, are shown.

### 2.3. Compression Tests

Compression tests were carried out according to the International Standard ASTM D3410 [27]. Specimens with a rectangular cross-section and a nominal width equal to 13 mm and a length of 150 mm were tested. The fixtures prescribed in the ASTM D3410 were used, to avoid specimen buckling and reliably measure the compression properties with an unclamped length of 10 mm. The strain was measured with a strain gauge (HBM 1-LY48-3/350) attached at the center of the unclamped length and the National Instruments NI 9237 system was employed to complete the Wheatstone Bridge and to properly amplify the strain signal.

**Figure 3:**
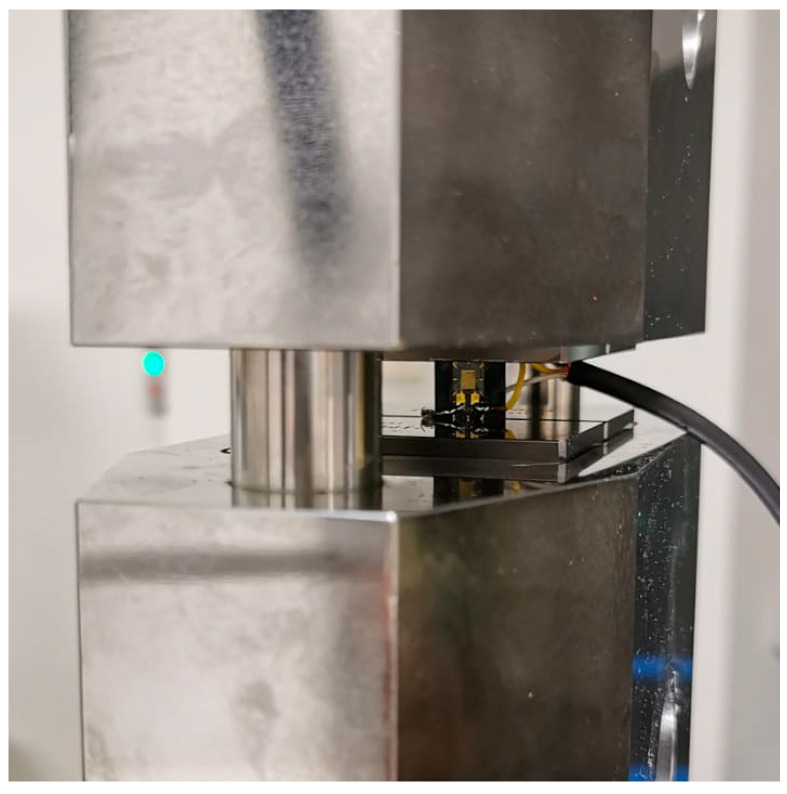
Compression tests: testing configuration.

Figure 3 shows the compression test setup. The unclamped specimen region and the strain gage used for measuring the strain are shown in Figure.

### 2.4. Shear Tests

**Figure 4:**
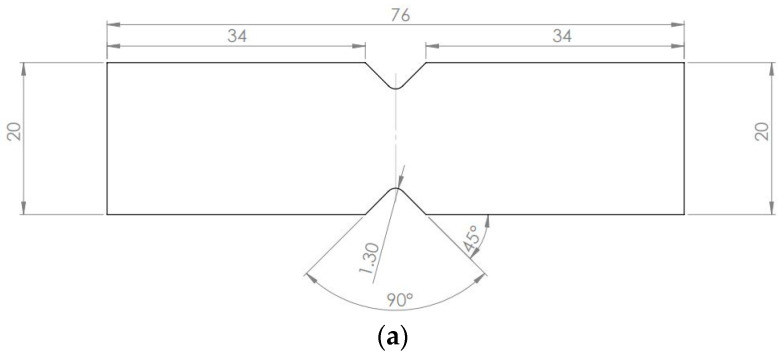

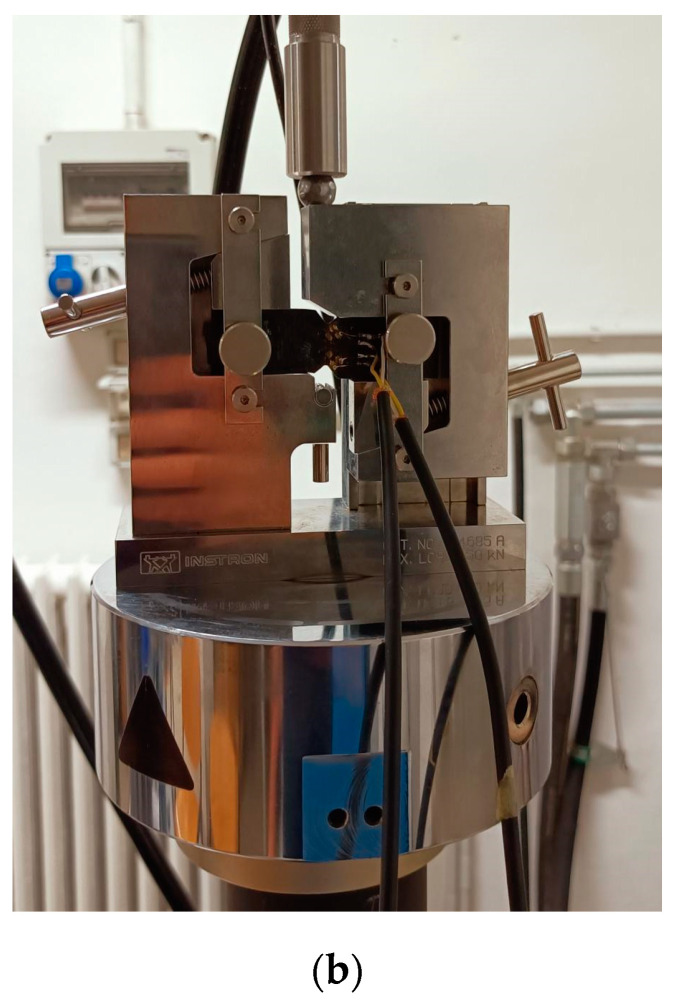
V-notched beam method for assessing the shear properties: (**a**) specimen geometry and strain gage location; (**b**) testing setup.

The shear properties of the two investigated composite laminates were experimentally assessed with the V-Notched Beam Method, according to the ASTM D5379 standard [28]. The specimen geometry is shown in Figure 4a. The strain was measured with two strain gauges (HBM 1-LY41-1.5/350, in a quarter bridge configuration) attached at 45° with respect to the loading axis, as close as possible to the middle of the specimen, but away from the notches. A National Instruments NI 9237 system was used for to complete the Wheatstone Bridge and to properly amplify the acquired signal An Instron 8801 testing machine, equipped with a 100 kN load cell, was used for the tests by imposing a crosshead displacement of 1 mm/min. Figure 4b shows the experimental setup with the attached strain gages.

### 2.5. Flexural Tests

**Figure 5:**
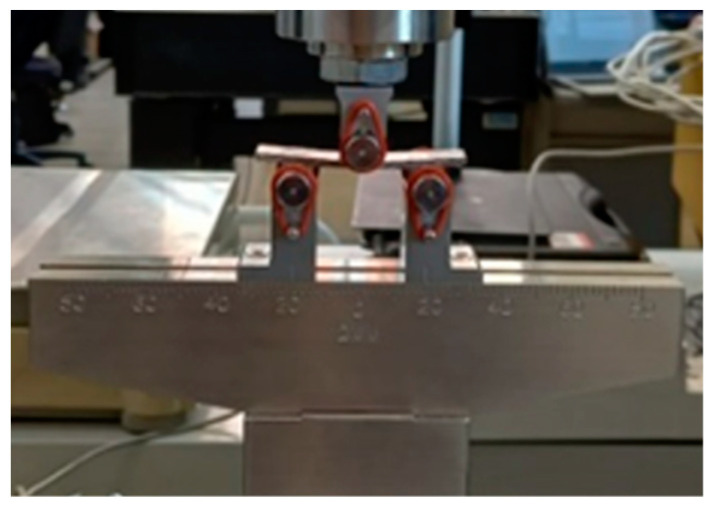
Flexural tests: testing configuration.

Flexural tests were carried out according to the ASTM standard D790 [29]. Rectangular cross-section specimens with a nominal width of 12.7 mm were tested. A support span of 31.6 mm, calculated as sixteen times the average thickness of the specimens, was used for the test (total length of 80 mm). The speed of the test, set to 0.8 $\frac{\mathrm{mm}}{\min}$, was selected according to the ASTM standard D790, based on the average thickness of the specimens and the support span. The flexural strain and stress were obtained with the equations $\varepsilon =\frac{6Dd}{L^{2}}$ and $\sigma =\frac{3PL}{2bd^{2}}$, respectively, where P is the load, L is the support span, b is the width of the specimen, d is the thickness of the specimen and D is the maximum deflection of the center of the beam, according to the ASTM standard and beam theory. The experimental setup is shown in Figure 5.

### 2.6. Finite Element Model

The experimental results were used to set a material model in a LS-Dyna environment. The goal was to provide a simple material model of the two tested composites which can be used for the design of the components. Even though the retained materials are woven composites, the material card *MAT_54, which assumes a Chang–Chang failure criterion, was chosen as it is one of the most commonly adopted for the simulation of composites [30,31]. As the fiber content in the two in-plane directions is the same, the 2WAY flag was activated. The failure criteria under tensile and compressive loads are the same in the two in-plane material directions.

For the elastic field, the software requires the definition of the Young’s moduli in the in-plane directions, E1 and E2; the Young’s modulus in the transverse direction, E3; the three shear moduli, G12, G13 and G23; and the three Poisson’s coefficients, v12, v13 and v23. Regarding the material strengths, *MAT_54 allows to define different strengths along the main material in-plane directions and to distinguish between the tensile and the compressive failure. In particular, the maximum longitudinal tensile strength, XT, the maximum longitudinal compressive strength, XC, the maximum transversal tensile strength, YT, the maximum transversal compressive strength, YC, and the in-plane shear strength, S, can be defined. It is worth noting that, on the contrary, elastic properties cannot be specified according to the tension or compression deformations.

The model was validated with the corresponding experimental results. Tests were replicated in LS-Dyna and the experimental material properties were considered. The laminate composites were modelled with four-node Belytschko–Tsay shell elements with a mesh size of 0.5 mm in all the tests. The four-node shell elements allow to obtain fast yet accurate results and, for this reason, are usually preferred when simulating structural components [30,31]. Eight integration points, one for each layer, were considered.

Given the simplicity of the tests, tensile and compressive tests were simulated with a single element model [32]. Displacement was imposed to two adjacent nodes, simulating the imposed displacement by the testing machine, while the counterposed nodes were constrained.

**Figure 6:**
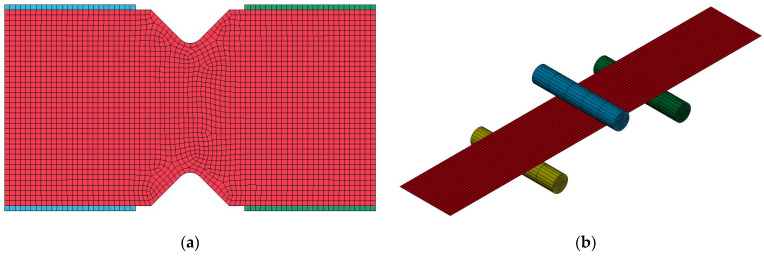
Numerical models of the V-notch test (**a**) and of the flexural test (**b**).

Figure 6 shows the numerical models of the V-notch test (Figure 6a) and of the flexural test (Figure 6b).

The V-notch test was simulated by considering rigid elements in contact with the specimen, that are the blue and green elements in Figure 6a. As in the experimental test, one side of the rigid element was constrained, while the other followed an imposed displacement law.

The three-point flexural test was simulated by means of rigid cylinders, as shown in Figure 6b. The upper cylinder moved, while the two supports at the bottom of the specimen were constrained. In order to simulate the elastic behavior of the material under flexural tests, two different models were considered and compared. In the first, material properties resulting from the compression tests were considered for the first four layers at the top of the specimen, which are subjected to compression in the flexural test. On the contrary, the material properties resulting from the tensile tests were considered for the second four layers at the bottom of the specimen, which are subjected to tensile load in the flexural test [33].

In the second model, the elastic properties calculated from the flexural tests were retained. Indeed, as observed in the literature, the flexural properties of composites can be consistently different from those obtained through standard tensile and compressive tests [34]. This discrepancy can be ascribed to the difference between the elastic tensile and compressive moduli, which is accounted for in the first model. In addition, a larger presence of resin in the upper and lower surfaces can consistently affect the flexural behavior [35]. On the contrary, the effect of these resin-rich areas on the elastic tensile and compressive moduli is limited.

Finally, it is worth noting that the results of this test, i.e., the specimen stiffness and the maximum force, are strongly sensitive to the thickness of the material. The mean value of the measured thickness was considered in the finite element models, with the thickness of the epoxy-based composite equal to 1.86 mm and that of the bio-based composite equal to 2.08 mm.

## 3. Results

In this section, the experimental results are commented and analyzed. In Section 3.1, the results of the tensile tests on the epoxy and the bio-based resins are reported. In Section 3.2, the tensile, compression, shear and flexural test results on the composite specimens are analyzed and compared. Finally, in Section 3.3, the experimental and numerical results are compared for the validation of the numerical model.

### 3.1. Tensile Tests on the Resins

**Figure 7:**
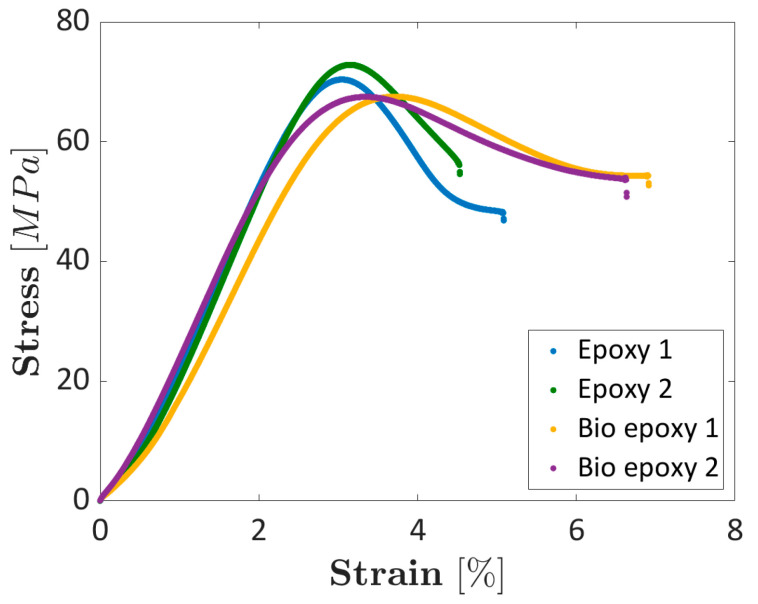
Stress–strain curves obtained by testing the epoxy and the bio-based resins.

Figure 7 compares the stress–strain curves obtained by testing the dogbone specimens made with the investigated epoxy and bio-based resins.

Figure 7 shows that the curves obtained by testing the same resin present a good repeatability, following a similar trend. The maximum stress is close to that provided by the resin supplier and is higher for the epoxy resin (in the range of (70–73) MPa with respect to the range of (67–68) MPa for the bio-based resin). The Young’s modulus is larger for the epoxy resin ((3029–3093) MPa with respect to that of (2633–2810) MPa for the bio-based resin), in agreement with the dataset provided by the resin supplier. On the other hand, the elongation to failure was found to be larger for the bio-based resin (in the range of (6.6–6.9)% with respect to the range (4.5–5.1)%), probably due to effect of the bio content and slight differences in the specimen preparation. Moreover, the small differences between the material parameters found experimentally and that provided by the resin supplier can be ascribed to the different types of tests, i.e., tensile tests in the present work and flexural tests carried out by the material supplier.

### 3.2. Quasi-Static Properties of the Investigated Composites

The results of the experimental activity on the investigated composites are here reported and commented on.

#### 3.2.1. Tensile Tests on Composite Laminates

**Figure 8:**
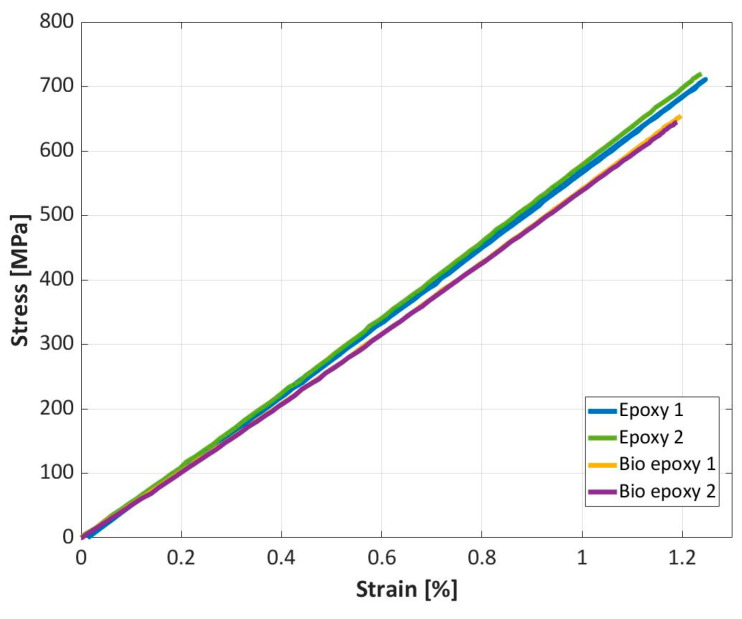
Stress–strain curves obtained by testing the epoxy and the bio-based resins.

Figure 8 compares the stress–strain curves obtained through tensile tests on the composite specimens made with the investigated epoxy and bio-based resins.

**Figure 9:**
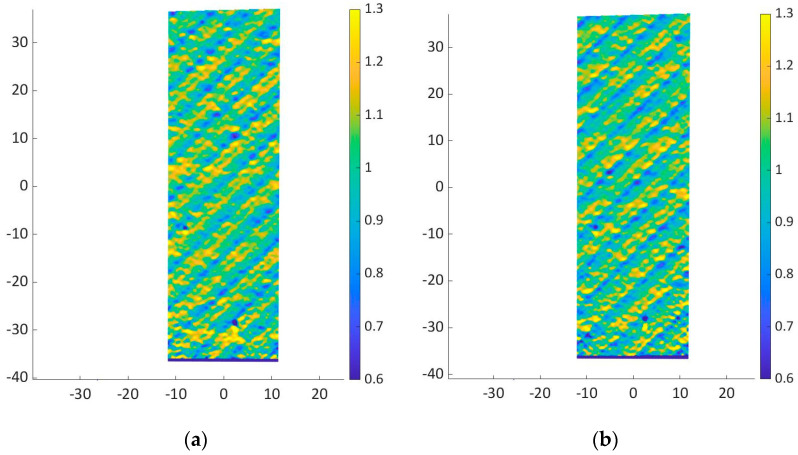

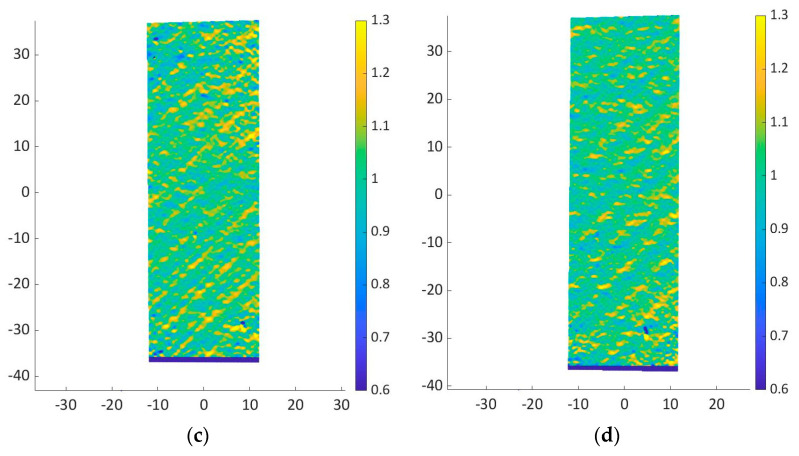
Strain map at 10 kN for specimen (**a**) epoxy-based composite 1; (**b**) epoxy-based composite 2; (**c**) bio-based composite 1; (**d**) bio-based composite 2.

The strain was calculated as the average value of the strains measured on the specimen surface through the DIC. Indeed, in woven composites, strains varied with the longitudinal or transversal tows of the texture. This can be appreciated by the strain maps reported in Figure 9. For a consistent comparison, the strains are mapped at the same applied load, that is, 10 kN.

As shown in Figure 8, the stress–strain curves are linear until failure occurs. Good repeatability can be observed. Regarding the elastic properties, in agreement with the results of the tensile tests on the resin specimens, the Young’s modulus of the epoxy-based composite is higher than that of the bio-based composite. In particular, the Young’s modulus of the epoxy-based composite is in the range of 57.1–58.4 GPa, while for the bio-based composite, the Young’s modulus is in the range 54.7–54.9 GPa. Additionally, the ultimate strength of the epoxy-based composite is higher than that of the bio-based composite, which can be again attributed to the higher properties of the epoxy resin, as shown in Section 3.1. The maximum strength of the epoxy composite is in the range of 711–720 MPa, consistently higher than that of the bio-based composite which ranges between 645 and 655 MPa.

The failure mode of all the specimens was found to be the same and within those considered valid in the ASTM Standard (failure mode LAT (Lateral at grip/Tab-Top)). The experimental scatter can be ascribed not to a variation in the failure mode, but rather to the typical scatter of experimental tests on the composite material, such as local weaknesses in the plate where specimens were cut.

#### 3.2.2. Compression Tests on Composite Specimens

**Figure 10:**
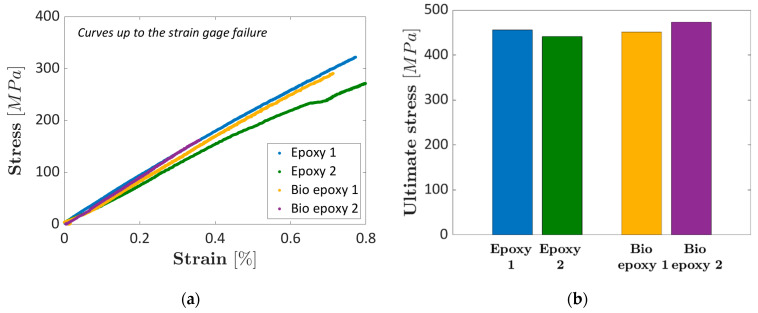
Compression tests on epoxy and bio-based composite specimens: (**a**) stress–strain curves obtained by testing the epoxy and the bio-based composites (the curves are truncated at the strain gage failure); (**b**) histogram showing the ultimate compressive stress.

Figure 10 shows the stress–strain curves obtained through compression tests on the composite specimens made with the investigated epoxy and bio-based resin. All the strain gauges failed before the specimen failure. Therefore, it was not possible to experimentally assess the maximum deformation at failure. Figure 10b compares the maximum compressive stress. All the failures occurred at the specimen center, with no differences between the failure modes of epoxy and bio epoxy composite specimens.

Figure 10a shows that the experimental curves are close to each other, but with quite a large scatter. The compressive modulus of elasticity, computed in the range (0.1–0.3)% according to the ASTM D3410, was found to be equal to 44 GPa and 41.2 Gpa for Epoxy 1 and Epoxy 2, respectively, and equal to 43.6 GPa and 46.5 GPa for the Bio epoxy 1 and the Bio epoxy 2, respectively. The compressive modulus, therefore, seems to be larger for the bio epoxy composite, but the range found by testing the epoxy and the bio epoxy composites overlaps. Accordingly, the difference can be ascribed mainly to the experimental scatter. A similar behavior can be found by comparing the ultimate stress, which is in the range (441–456) MPa for the epoxy composite and (451–473) MPa for the bio-based epoxy. The difference is not significant even for this parameter, with the two ranges overlapping. Due to the almost linear trend found experimentally in Figure 10a, a reasonable estimation of the maximum deformation at failure can be obtained by dividing the maximum stress by the compressive elastic modulus. The deformation to failure was estimated to be in the range (1.03–1.07)% for the epoxy composite and in the range (1.02–1.03)% for the bio-based epoxy composite, with no differences between the investigated composites. Even for this load type, the failure mode was found to be the same, with no differences between the epoxy and the bio-based composites. The failure mode corresponds to the BGM (Brooming-Gauge-Middle) failure mode reported in the ASTM Standard D3410.

Accordingly, it can be concluded that the bio-based resin has the same compressive behavior as the epoxy resin.

#### 3.2.3. Shear Tests on Composite Specimens

**Figure 11:**
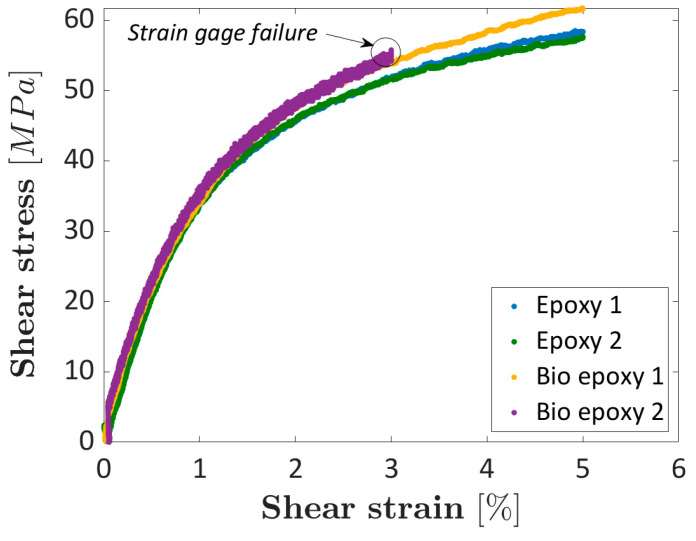
Shear stress–strain curves obtained by testing the investigated epoxy and the bio-based composites (the curves are truncated at 5% strain, according to ASTM D5379 standard).

In this section, the shear properties are compared. The shear strain was computed according to the indications in the ASTM D5379 standard, starting from the acquired strain gauge signal. Figure 11 compares the stress–strain curves for the investigated composite specimens. According to the ASTM D5379 standard, for specimens failing above 5% strain, 5% strain has to be considered as the ultimate strain and the ultimate stress corresponds to the stress at a strain equal to 5%. Strain gauges in specimen Epoxy bio 2, however, failed before 5% strain. For this specimen, the ultimate stress and strain cannot be computed. The curves plotted in Figure 11 were truncated at 5% strain, according to the ASTM D5379 standard.

Figure 11 shows that the curves for the epoxy and the bio-based composites follow a similar trend. The shear modulus of elasticity is slightly larger for the epoxy composite ((4090–4135) MPa for the epoxy composite and (3719–3840) MPa for the bio-based composite). The difference is, however, small and close to the scatter between the shear modulus of elasticity computed for the same material. On the other hand, the ultimate strength is larger for the bio-based composite, in the range of (57.6–58.5) MPa for the epoxy composite and equal to 61.7 MPa for the bio-based composite. Epoxy bio 2 specimen, whose curve is truncated due to the strain gauge failure, follows the same trend of Epoxy bio 1 specimen, thus confirming the trend found for the bio-based epoxy composite, and a similar ultimate strength can be reasonably expected. The failure mode of the tested specimens was analyzed and corresponds to the failure mode MGN—Multimode-Gauge-Between Notches, according to the ASTM Standard D5379, since it occurred in the gauge cross-section and it started from the specimen notch. The fracture surfaces of the epoxy and the bio-based resins, accordingly, show no differences. To conclude, the shear modulus of elasticity was found to be larger for the epoxy composites, which, on the other hand, showed a smaller tensile strength. However, in both cases, the difference is small. The ultimate strain, on the other hand, was found to be above 5% for both investigated composite types.

Considering the slightly higher properties of the epoxy resin, as shown in Section 3.1, the higher strength of the bio-based composite suggests that the bonding between the carbon fibers and the bio-based resin is better than that between the carbon fabric and the epoxy resin, thus highlighting another possible advantage of the bio-based composite. However, further investigations at the microscopic level are required in this regard.

#### 3.2.4. Flexural Tests on Composite Specimens

**Figure 12:**
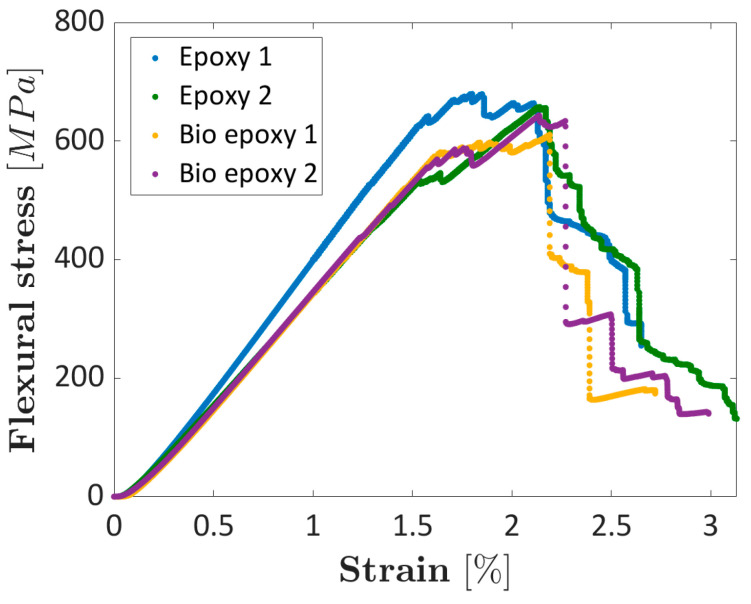
Stress–strain curves obtained through flexural tests.

The stress–strain curves related to the flexural tests are reported in Figure 12, which show that both bio-based epoxy and traditional epoxy composite laminates exhibit a similar general trend.

**Figure 13:**
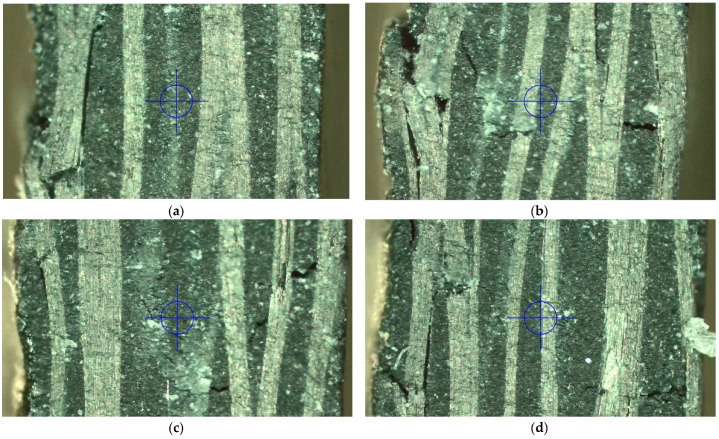
Fracture surface of the flexural specimens (compressed side is on the left of the figures): (**a**) Epoxy-1; (**b**) Epoxy-2; (**c**) Bio-epoxy-1; (**d**) Bio-epoxy-2.

The epoxy composites showed a maximum flexural stress of (657–659) MPa and a flexural modulus of (37.7–45.6) GPa. The bio-based composites showed a maximum flexural stress of (610–643) MPa and a flexural modulus of (39.3–39.4) GPa. It can be noted that, while for the epoxy-based composite, the flexural modulus is very similar to that obtained in the compression test, indicating that the compressive modulus governs the flexural behavior, in the bio-based composite, the flexural modulus is consistently lower than that calculated in the compression test. This could be due to the higher amount of resin present in the bio-based composite. Indeed, the presence of resin-rich areas in the upper and lower surfaces can consistently affect the flexural behavior [35]. The fracture zones are reported in Figure 13 (Figure 13a,b for Epoxy-1 and Epoxy-2, respectively, whereas Figure 13c,d for Bio-epoxy-1 and the Bio-epoxy-2, respectively). The compressed side is on the left of the figures.

Figure 13 shows that the longitudinal tows in epoxy laminates present fractures only in the compressive zone. Indeed, the compressive strength was consistently lower than the tension strength. Cracks are however present in the transversal tows, as it can be appreciated in Figure 13b. On the other hand, in the bio-based laminates, the longitudinal tows presented fracture in both the tensile and compressed sides. Additionally, in this case it can be argued that the first fracture likely occurred in the compressed side, as the compressive strength was consistently lower than that of tension.

### 3.3. Finite Element Analysis

**Figure 14:**
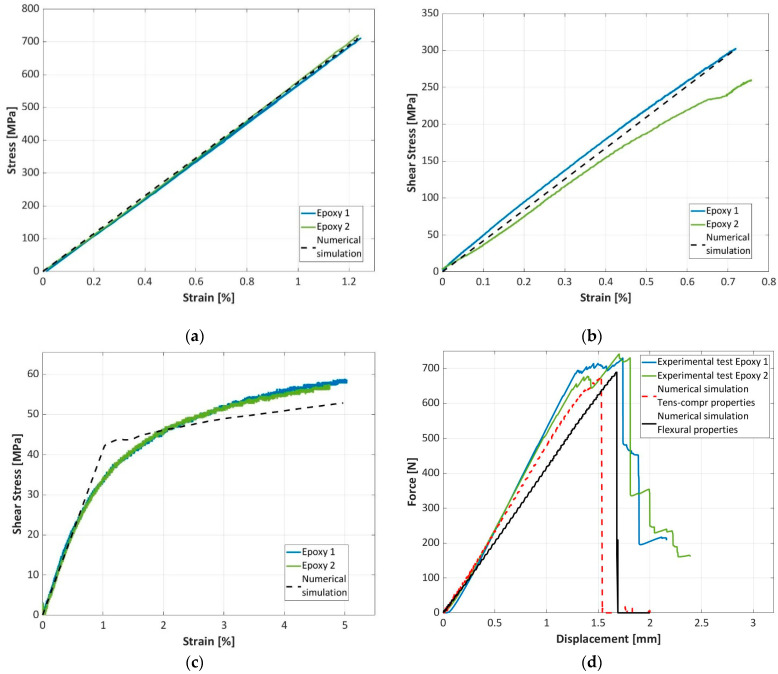
Comparison of the experimental and numerical results for the epoxy-based composite: (**a**) tensile test; (**b**) compressive test; (**c**) Iosipescu test; (**d**) flexural test.

**Figure 15:**
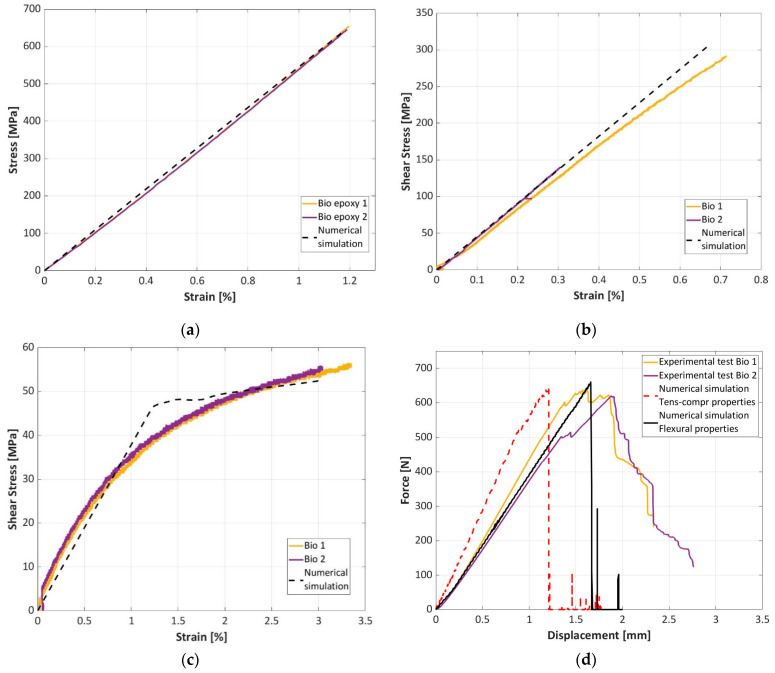
Comparison of the experimental and numerical results for the bio-based composite: (**a**) tensile test; (**b**) compressive test; (**c**) Iosipescu test; (**d**) flexural test.

In this section, material models of the investigated epoxy-based and bio-based composites in the LS-Dyna environment are validated using the experimental results shown in Section 3. Figure 14 compares the experimental and the numerical curves for the epoxy-based composite, while Figure 15 compares the experimental and numerical curves for the bio-based composite. In Figure 14 and Figure 15, the tensile test (Figure 14a and Figure 15a), the compressive test (Figure 14b and Figure 15b), the shear test (Figure 14c and Figure 15c) and the flexural test (Figure 14d and Figure 15d) are considered. For the tensile, compressive and V-notched tests, the comparison is reported in terms of stress and strain, while for the flexural test, the force displacement curves are compared.

In general, the material model captures the mechanical behavior of the tested materials well. The linear trends of the tensile and compressive tests are well replicated by this simple material model. However, the material model also captures the nonlinear behavior of the materials well when in-plane shear load is applied. In this regard, it is worth mentioning that the shear stress and the shear strain were calculated as an average of the shear stresses and strains of the elements corresponding to the notched section. The nonlinearity was accounted for through the ALPHA parameter of the material card *MAT_54 [36,37], assumed to be equal to 2.4 × 10^−7^.

Finally, regarding the flexural test, the force–displacement curves are well replicated only by the finite element model that accounts for the flexural properties as determined from the flexural tests. In particular, the stiffness of the finite element model that assumes tensile and compressive properties in the bottom and upper layers, respectively, well replicates the experimental results only in the case of the epoxy-based composite. On the contrary, in the case of the bio-based composite, a consistently higher stiffness is obtained in finite element model.

Indeed, as observed in Section 3.2, the tensile modulus of the epoxy-based composite is higher than that of the bio-based composite; that is, the mean value is 57.8 GPa for the epoxy-based and 54.8 GPa for the bio-based composites. On the contrary, the Young’s modulus in compression is slightly higher for the bio-based composite, the mean value being 45.5 GPa, with respect to that of the epoxy-based composite (mean value of 42.6 GPa). Therefore, in the flexural tests, the elastic properties of the two composites are similar. However, the bio-based composite is consistently thicker than the epoxy-based and so the numerical model of the bio-based specimen is stiff.

This suggests that the approach that assumes tensile and compressive properties in the bottom and upper layers, respectively, is not sufficient to replicate the material behavior under flexural tests. The difference can be ascribed to the larger presence of resin in the bio-based composite. Indeed, each layer of the bio-based composite is 0.263 mm thick, while in the epoxy-based composite, the lamina thickness is 0.245 mm. The larger presence of resin has limited influence on the elastic properties in pure tensile and compressive loading condition which are strongly governed by the fabric texture, but it consistently affects the flexural elastic behavior, particularly reducing the global stiffness.

Both the models also replicated the maximum force with good accuracy, while they were not able to capture the post-peak behavior. Failure has likely started from the upper compressed side, as the compressive strength of both materials is consistently lower than that of tension, as observed in Section 3.2.4. Thereafter, the failure progressively proceeded towards the lower layers, until final fracture occurred and the load dropped. As the crack progressively propagated from the compressed side, friction between the cracked surfaces was also present. However, the shell elements of the finite element model cannot replicate this behavior.

In conclusion, despite its simplicity, the retained material model can be reliably used in the design of components made with the investigated materials, as it is able to replicate the strengths of the materials well. Furthermore, in case of flexural loading conditions, compressive and tensile properties should be properly retained in the FE model, as both the elastic and the strengths properties consistently vary with the loading conditions in the two materials.

## 4. Conclusions

In the present paper, the quasi-static mechanical properties of composite specimens (eight layers carbon fiber fabric composite) made with commercially available epoxy resins and bio-based epoxy resins (31% of bio content) are compared. Tensile tests on the two resins and tensile, compression, shear and flexural tests were carried on specimens cut from the plate manufactured with the two investigated resins. A finite element model was also calibrated with the results of the quasi-static tests.

Table 2 summarizes the obtained results.

The following conclusions can be drawn:

1. The stress–strain curves obtained through tensile tests are linear until failure occurs, with good repeatability among the tests. The Young’s modulus (57.1–58.4 GPa for the epoxy and 54.7–54.9 GPa for the bio-based) of the epoxy-based composite was found to be higher than that of the bio-based composite. The maximum strength of the epoxy composite was found in the range of 711–720 MPa, consistently higher than that of the bio-based composite, which ranged between 645 and 655 MPa. This agreed with the results of the tensile tests on the resins.
2. The stress–strain curves obtained through compression tests were found to follow a similar trend, with no significant differences between the compressive moduli. Similarly, the experimental shear strength and the deformation to failure ranges overlapped, with no differences between the investigated composites.
3. The epoxy and bio-based composites shear stress–strain curves followed a similar trend, with the shear modulus of elasticity slightly larger for the epoxy composite. On the other hand, the ultimate strength was larger for the bio-based composite. Accordingly, even if slight differences were found, it can be concluded that the epoxy and the bio-based composites show similar shear properties.
4. Flexural tests have shown that the compressive side governs the mechanical behavior. The flexural modulus was found to be similar to that obtained in the compression test in the case of the epoxy-based composite. For the bio-based composite, the flexural modulus was slightly lower than the values obtained in the compression test. This could be due to the higher amount of resin of the bio-based composite. The bio-based composite also showed fractures in the tensile side, which likely occurred in a second phase.
5. The material parameters of the epoxy-based and bio-based composites were identified for finite element simulation in the LS-Dyna environment with *MAT_54. The identified parameters well replicated the experimental behavior under different loading conditions and can be used for the simulation of complex components or structures.

To conclude, the analyses carried out in this paper have proven that, even if slight differences in the quasi-static mechanical properties of the investigated epoxy and bio-based composites were found, especially for the tensile properties, bio-based composites with a bio content up to 31% can be reliably employed. Indeed, the slight experimental differences are within the scatter range typical of composite materials, proving moreover that a resin with a bio-content up to 31% does not induce chemical modifications in the structure of the composite, thus not affecting its structural integrity. Accordingly, bio-based composites with a bio content up to 31% could potentially replace less sustainable epoxy composites currently widely adopted in many structural applications, lowering their carbon footprint.

## Figures and Tables

**Table 1:** Comparison of the mechanical properties of the two investigated resins [19,20].

Material	Density (g/cm^3^)	Young’s Modulus (MPa)	Strength (MPa)
Epoxy resin	1.14	3000	68.5
Bio-epoxy resin	1.12	2790	65

**Table 2:** Mechanical properties of the epoxy-based and bio-based composites. Means values and standard deviations are reported.

Property	Epoxy-Based Composite	Bio-Based Composite
Density (g/cm^3^)	1.41	1.41
Tensile Young’s modulus (GPa)	57.8–0.85	54.8–0.14
Poisson ratio	0.045–0.0016	0.047–0.0004
Tensile strength (MPa)	716–5.65	650–7.07
Tensile strain at break (%)	1.25–7 × 10^−4^	1.195–7 × 10^−4^
Compressive modulus (GPa)	41–3.0	45–2.1
Compressive strength (MPa)	450–10	460–16
Compressive strain at break (%)	−1.052–2.5 × 10^−4^	−1.026–1.2 × 10^−4^
In-plane shear modulus (GPa)	3.46–0.11	3.0–0.013
In-plane shear strength (MPa)	57–0.7	61.2
Flexural modulus (GPa)	41.7–5.6	39.3–0.1

## Data Availability

Experimental data requests can be directed to carlo.boursier@polito.it.
